# The Epidemiology of Infectious Diseases Meets AI: A Match Made in Heaven

**DOI:** 10.3390/pathogens12020317

**Published:** 2023-02-15

**Authors:** Ankur Bothra, Yongguo Cao, Jiří Černý, Gunjan Arora

**Affiliations:** 1 National Institute of Allergy and Infectious Diseases (NIAID), Bethesda, MD 20892, USA; 2 College of Veterinary Medicine, Jilin University, Changchun 130062, China; 3 Centre for Infectious Animal Diseases, Faculty of Tropical AgriSciences, Czech University of Life Sciences Prague, 165 00 Prague, Czech Republic; 4 Section of Infectious Diseases, Yale School of Medicine, Yale University, New Haven, CT 06519, USA

## An Opinion

Infectious diseases remain a major threat to public health [1,2,3]. This Special Issue on the Epidemiology of Infectious Disease will cover studies related to the emergence, transmission, and containment of infectious diseases, including new research showing potential therapeutic interventions. This Issue will encompass viral, bacterial, and parasitic diseases with an emphasis on emerging research areas such as modeling, clinical studies, longitudinal cohort, and case–control studies, systems biology approaches, artificial intelligence (AI), machine learning, and other molecular and immunological studies [4,5,6].

AI and machine learning can be employed to study complex interactions between different biological systems, such as signaling pathways and metabolic networks, to advance our understanding of various biological phenomena and improve the diagnosis and treatment of diseases [5,7,8]. These technologies have the potential to significantly impact biological research in a variety of areas, including infectious diseases and epidemiology, as highlighted in the Special Issue of the MDPI journal *Pathogens* entitled “Papers on the Epidemiology of Infectious Diseases”.

AI and machine learning can be used to analyze large datasets, such as genomic data, to identify patterns and trends relevant to the understanding and treatment of infectious diseases [9,10,11,12]. For example, machine learning algorithms have been utilized to identify potential drug targets for SARS-CoV-2, which causes COVID-19 [13,14]. In addition, AI and machine learning can be employed to predict the likelihood of certain outcomes, such as the spread of a disease, based on historical data and by analyzing datasets generated by epidemiological studies. This can aid epidemiologists in preventing or mitigating outbreaks of infectious diseases, such as influenza and HIV.

AI can also be utilized to build predictive models that help researchers understand the relationships between different variables, such as gene expression and disease risk, interactions between pathogens and host organisms at the molecular level, and complex molecular interactions within biomolecules. Examples of the use of AI in biological research include AlphaFold [15,16], which can predict the secondary and tertiary structure of proteins with a high level of confidence [17,18], and DeepMind, which analyzes images of cells or tissues to identify specific features or patterns relevant to research.

An application that recently received media attention is AI’s capability in processing natural languages. In this regard, Open AI’s chatbot, named ChatGPT, can process natural language text and can be used to perform complex analysis and help non-English-speaking epidemiologists to draft articles. ChatGPT can provide the definitions of scientific terms, generate prevalence and risk factor maps of any disease, and so on. These efforts can revolutionize biological science research, but the output from such AI platforms needs to be verified, especially in many social, economic, behavioral, and epidemiological studies that deal with large datasets. Therefore, it demands the assessment of security issues and other challenges that arise in the use of ChatGPT. The validation of AI-based research using standard conventional methods is critical to check the flow of misinformation. The goal for the future development of such AIs is to train them to use relevant data sources; otherwise, we will jump from the post-factual age directly into the non-factual age. Anecdotally, these tools have been found to provide non-existing references in digital epidemiology.

Overall, the use of AI and machine learning in biological research, including epidemiology, has the potential to speed up the research process, improve accuracy and precision, and allow researchers to tackle more complex questions. We encourage our readers to use AI and publish their research in the MDPI journal *Pathogens*.
